# Sleep-Wake Regulation and Its Impact on Working Memory Performance: The Role of Adenosine

**DOI:** 10.3390/biology5010011

**Published:** 2016-02-05

**Authors:** Carolin Franziska Reichert, Micheline Maire, Christina Schmidt, Christian Cajochen

**Affiliations:** 1 Centre for Chronobiology, Psychiatric Hospital of the University of Basel, Wilhelm Klein-Strasse 27, Basel 4012, Switzerland; mi_maire@hotmail.com (M.M.); christian.cajochen@upkbs.ch (C.C.); 2 Transfaculty Research Platform Molecular and Cognitive Neurosciences, University of Basel, Birmannsgasse 8, Basel 4055, Switzerland; 3 Cyclotron Research Centre, University of Liège, Allée du 6 Août n°8, Liège 4000, Belgium; christina.schmidt@ulg.ac.be

**Keywords:** circadian, sleep homeostasis, adenosine, working memory, cognition

## Abstract

The sleep-wake cycle is regulated by a fine-tuned interplay between sleep-homeostatic and circadian mechanisms. Compelling evidence suggests that adenosine plays an important role in mediating the increase of homeostatic sleep pressure during time spent awake and its decrease during sleep. Here, we summarize evidence that adenosinergic mechanisms regulate not only the dynamic of sleep pressure, but are also implicated in the interaction of homeostatic and circadian processes. We review how this interaction becomes evident at several levels, including electrophysiological data, neuroimaging studies and behavioral observations. Regarding complex human behavior, we particularly focus on sleep-wake regulatory influences on working memory performance and underlying brain activity, with a specific emphasis on the role of adenosine in this interplay. We conclude that a change in adenosinergic mechanisms, whether exogenous or endogenous, does not only impact on sleep-homeostatic processes, but also interferes with the circadian timing system.

## 1. Introduction

The two-process model of sleep-wake regulation was initially proposed in 1982 by Borbély and has been continuously refined ever since [1,2]. Overwhelming evidence confirmed that mainly two processes, a sleep-homeostatic and a circadian process, regulate quantity, quality and timing of sleep and wakefulness. Furthermore, empirical observations showed that sleep-homeostatic and circadian mechanisms interact: The influence of the circadian timing system, as mirrored at a genetic [3], electrophysiological [4], blood-oxygen-level dependent (BOLD) [5] and behavioral level [6] is dependent on the status of the sleep homeostat. The neuro-pharmacological mechanisms underlying this interaction are currently investigated.

Here, we focus on the role of adenosine, a neuro-modulator ubiquitously present in widespread cerebral networks [7,8]. Based on extensive evidence, this nucleoside has been thought to be one of the so-called sleep-factors [9], *i.e.*, substances which increase during wakefulness and decrease during time spent asleep, particularly during deep sleep. These sleep-factors (see [9] for overview) are thus thought to track homeostatic sleep pressure and to mediate its effects ranging from metabolism and immune functions to highly complex cognitive behaviors. In this review, we summarize evidence that adenosine is implicated in the sleep pressure-dependent modulation of circadian mechanisms.

The impact of sleep pressure on working memory (WM) performance and its underlying cerebral correlates has repeatedly been investigated (see [10] for overview). Studies indicate that WM functions do not simply decline with rising sleep pressure, but are differentially regulated by factors interacting with the sleep homeostatic process, such as inter-individual differences (e.g., in genetic polymorphisms [11,12,13,14], age (for overview, see [15])) or time of day ([16,17,18,19,20,21], but see [22,23]). After summarizing evidence about circadian and sleep-homeostatic, in particular adenosinergic mechanisms on electroencephalographic (EEG), hormonal and subjective sleepiness data, we will specifically review the current knowledge about the regulation of WM performance and the underlying neuronal mechanisms.

## 2. Sleep-Wake Regulation: Concepts and Empirical Evidence

### 2.1. Sleep-Wake Regulation at a Conceptual Level

The timing, duration, and quality of sleep and wakefulness are regulated by the combined action of two processes [1]. The sleep homeostatic process can be basically described as a rise of sleep pressure during wakefulness and its dissipation during sleep, as measured by slow electroencephalographic (EEG) activity [1,24]. The term “homeostasis” refers to the compensatory facilitation of deep, continuous, and long sleep episodes when sleep is initiated after a long episode of wakefulness [25]. Neuropharmacologically, several substances, so-called sleep factors, have been identified, mediating the dynamics of sleep homeostatic effects during wakefulness and sleep [9]. The specific function of sleep homeostatic mechanisms in the brain has been mainly discussed in terms of energy restoration and cellular defense [9] as well as synaptic plasticity [26].

The second process refers to circadian oscillations (lat. circa = about, dies = day), which superimpose a nearly 24-h pattern on the sleep-wake cycle: In diurnal species, the circadian system actively promotes wakefulness during the biological day, while it promotes sleep during the biological night, *i.e.*, during phases of melatonin secretion by the pineal gland [27,28]. This rhythm is triggered and adjusted to the external light-dark cycle by light inputs to the brain’s main circadian pacemaker, the suprachiasmatic nuclei (SCN) of the anterior hypothalamus [29]. Within the SCN, a genetic clockwork determines the endogenous rhythm by a self-sustaining feedback loop with a duration of nearly 24 h [3]. The synchronization to the external light-dark cycle is mainly based on the ocular perception and transduction of environmental light information to the SCN [30]. This general mechanism to synchronize with the rhythm of environmental signals, so-called zeitgebers, is evolutionarily highly conserved and can be observed in almost all species [31].

**Figure 1:**
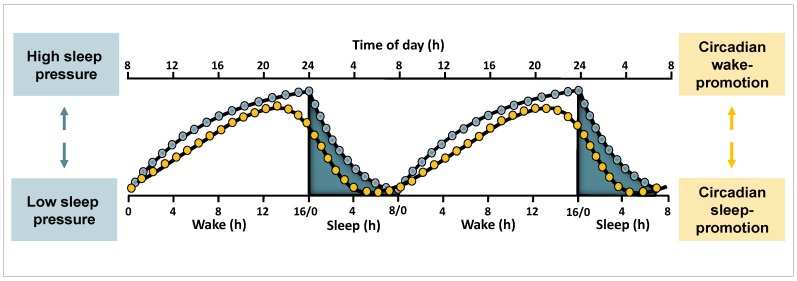
Illustration of human sleep-wake regulation by circadian and homeostatic mechanisms under entrained conditions. Under entrained conditions wakefulness is scheduled to daytime, and sleep to the biological night. The homeostatic sleep need (**blue**) increases during wakefulness and declines during sleep. Circadian oscillations (**yellow**) promote wakefulness during the day and sleep during the night, and are relatively independent of prior sleep-wake history. Figure and legend adapted from a previous publication [40]. Reproduced with permission.

Along the 24-h cycle, circadian and sleep homeostatic mechanisms act either in synchrony or in opposition to each other (Figure 1). When wakefulness of diurnal organisms is scheduled to occur during daytime and sleep during nighttime (*i.e.*, under so-called entrained conditions), circadian arousal-promoting mechanisms oppose rising sleep pressure levels during daytime [27]. This opposing action enables a consolidated episode of wakefulness under accumulating sleep need [28]. With the onset of melatonin secretion in the late evening hours, the circadian wake-promoting impact breaks down and the “gate for sleep” opens [32]. Together with high sleep pressure levels, this time can be considered as an optimal window for sleep initiation. Towards the end of a night-sleep episode, when sleep pressure has mostly dissipated, sleep is presumably maintained due to active circadian sleep-promoting mechanisms [27].

Overall, both circadian and sleep homeostatic mechanisms contribute to consolidated wake and sleep bouts under entrained conditions. Consequently, disruption of the interplay of both processes, for instance, due to shift work or travelling across time zones, reduces sleep and wake quality. At several behavioral and physiological levels, the impact of circadian modulations on sleep and wakefulness crucially depends on sleep pressure levels [6]. When sleep pressure is at low levels, the circadian arousal peak in the late evening hours is particularly pronounced [16,21], while typical circadian nighttime troughs in cognitive performance have been shown to be enhanced under high sleep pressure [16,17,21].

### 2.2. Investigating Circadian and Sleep Homeostatic Mechanisms

Several laboratory protocols have been developed to investigate the influence of circadian and sleep homeostatic mechanisms on human behavior and physiology. The most sophisticated design is the so-called forced desynchrony protocol. In such a study, participants are separated from the natural environment for several weeks and live according to a specific sleep-wake cycle. This artificial sleep-wake cycle corresponds to the usual 1:2 ratio of sleep and wakefulness, but is considerably longer or shorter than the regular 24-h cycle (e.g., [16,17]). As the circadian system is not able to keep track to the imposed environmental light-dark cycle, it starts to follow its own rhythm and begins to free-run. Furthermore, sleep and wakefulness occur systematically at differential times of the biological day or night and are desynchronized from the endogenous circadian rhythm. The influence of differential sleep pressure levels can thus be assessed at virtually all circadian phases, or, conversely, circadian influences can be measured under differential sleep pressure conditions. Consequently, a forced desynchrony protocol allows one to investigate the interaction between circadian and sleep homeostatic processes and to quantify their separate contribution to output parameters, such as sleep and waking electroencephalography (EEG) or cognitive performance.

A less time-consuming way to study the impact of differential sleep pressure levels at the same circadian phase is the implementation of a so-called constant routine protocol with a duration of more than 24 h. This design requires participants to stay continuously awake while the influence of potential zeitgebers such as light, body posture, meal intake, or sleep and wakefulness is kept constant [33]. The protocol was originally developed to investigate unmasked circadian rhythms. When extending wakefulness to more than 24 h, it also enables one to assess dependent variables at the same circadian phase under differential sleep pressure levels. However, it has to be taken into account that sleep deprivation (SD) *per se* might delay circadian phase position [34]. Additionally, a separation of circadian and sleep homeostatic influences is not possible, as a certain level of sleep pressure does not systematically occur at all circadian phases.

To control for this confound, multiple-nap protocols (NP) have been developed, in which regularly scheduled naps serve to keep homeostatic sleep pressure at a rather low level throughout the 24-h cycle. This allows the studying of the circadian course of several waking functions without the confounding rise in sleep pressure (e.g., [22,23,35,36,37]). Importantly, the regularly scheduled sleep episodes further enable one to assess circadian variations in differential sleep features [32,38,39]. A disadvantage is that the fragmentation of sleep prevents ultradian processes requiring long and continuous sleep-episodes. Nonetheless, a combination of a constant routine and an NP appears to be a useful alternative to the much more laborious forced desynchrony protocol.

### 2.3. Circadian and Sleep Homeostatic Regulation of Sleep and Waking Functions

#### 2.3.1. Circadian and Homeostatic Regulation of Sleep Features

Various sleep features are affected by circadian and sleep homeostatic mechanisms (e.g., [16,27,38,39,41,42]). Slow-wave sleep (SWS) duration [16] and NREM (non rapid-eye movement) sleep spectral power in the range of 0.7–4 Hz [42] mirror the dynamics of homeostatic sleep pressure [2], particularly in frontal areas [43]. These features are more pronounced if more time is spent awake before initiation of sleep, and decrease over the course of a sleep episode. Additionally, NREM EEG power density in the range of 12–16 Hz (sigma activity) shows a sleep homeostatic pattern as well, but is also strongly modulated by circadian phase [42].

**Figure 2:**
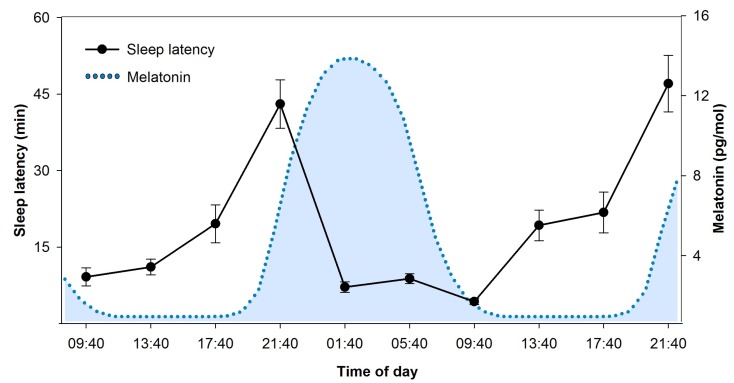
Time course of sleep latency over the 24-h cycle in a 40-h nap protocol. Sleep latency to sleep stage 1, assessed during regular naps of 80 min (NP, see [49] for details), shows a striking circadian pattern. Longest durations occur in the evening hours shortly before habitual bedtime and mirror highest levels of circadian wake promotion at the end of the biological day [27]. Shortest durations were measured during the biological night, which is illustrated by the blue dotted curve of melatonin secretion. Melatonin was analyzed in saliva samples collected in the same study [12,49] and modelled according to algorithms published in [50].

On the other hand, sleep latency (Figure 2) and sleep efficiency for example follow a clear-cut circadian pattern. They mirror the course of circadian arousal promotion, with difficulties to initiate and maintain sleep during daytime, specifically at the end of the biological day [27,39]. During the late evening hours at the end of a day, circadian wake-promotion reaches peak-levels (see Figure 1). Accordingly, this time window has been labelled as the “wake-maintenance zone” [44]. Similarly, peak levels of active circadian sleep promotion in the early morning (see Figure 1) have been proposed to be mirrored in prominent circadian peaks of REM (rapid-eye movement) sleep duration [41,45].

Generally, it should be noted that a strong circadian or homeostatic control of a specific sleep feature might not be understood as exclusive, but rather as a predominance of one of the two sleep-wake regulatory mechanisms under specific conditions. For instance, sleep latency is shortened under high sleep pressure [46,47], and sleep efficiency decreases according to time spent asleep [16]. Furthermore, REM sleep duration is modulated by time spent asleep, in a circadian phase-dependent manner [41]. Finally, the core marker of NREM sleep homeostasis, slow-wave activity (SWA), exhibits a “small but significant” [41] circadian variation [48]. Taken together, these observations strengthen the assumption of an inherent connection between circadian and sleep homeostatic mechanisms in the regulation of sleep features.

#### 2.3.2. Circadian and Homeostatic Regulation of Waking Functions

Circadian and homeostatic profiles have repeatedly been observed in several waking functions, ranging from waking EEG, to behavioral performance, and in both subjective and objective measures of sleepiness. For instance, alpha activity (8–12 Hz) decreases [51], and performance deteriorates, with time spent awake [16,17,18]. Similarly, frontal EEG delta activity (1–4.5 Hz) increases [51], and subjective sleepiness rises continuously, if more time is spent awake [16].

**Figure 3:**
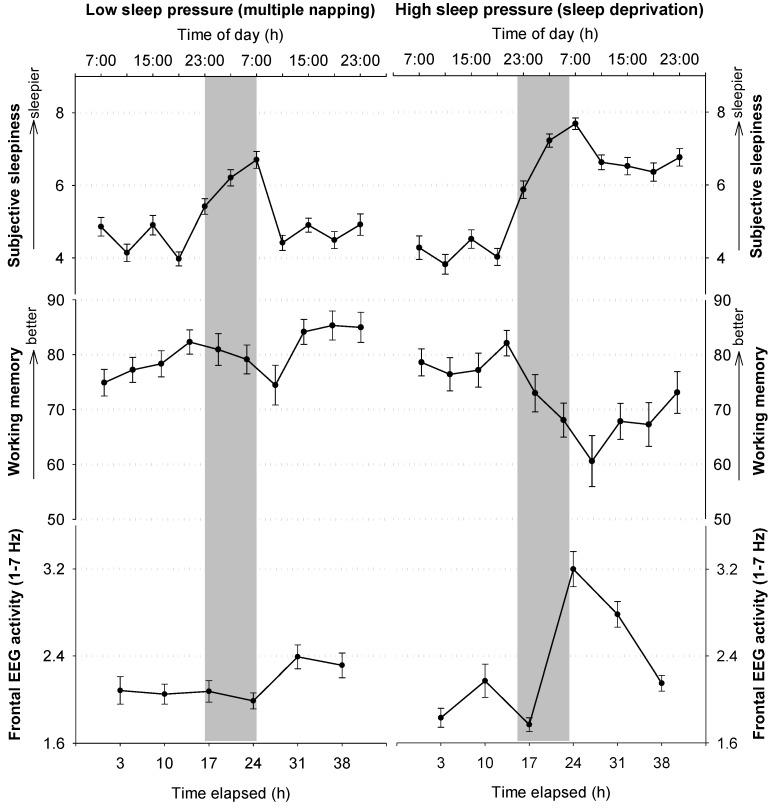
Circadian and homeostatic influences on subjective sleepiness, WM performance, and waking EEG. Values assessed during a low sleep pressure condition (NP) are depicted on the left panel, and mirror circadian influences under rather low sleep pressure conditions. On the right side, the impact of rising sleep pressure during night- and daytime is illustrated, as these values were assessed during a SD of 40 h. The grey bars indicate nighttime. Subjective sleepiness was assessed by a questionnaire (Karolinska Sleepiness Scale [53]), WM performance by an n-back task (depicted is the percentage of hits). The waking EEG was analyzed over three frontal derivations (F3, F4, FZ). Please see [12,49] for further information on data assessments and study protocol.

Most of these measures are as well affected by circadian phase. Generally, the impact of circadian phase has been shown as nighttime trough in waking EEG alpha activity (8–12 Hz; [51]) and cognitive performance [16,17,18]. Additionally, sleepiness is enhanced during nighttime, both subjectively (Figure 3; [16]) and objectively, as measured by electrooculographic slow-rolling eye movements [52].

Finally, both circadian and homeostatic mechanisms act in a combined manner on waking quality. The typical interaction of these processes can be nicely observed during SD (see Figure 3). During the first day, that is, under usual sleep pressure levels, frontal low EEG activity, sleepiness, well-being and performance are relatively stable. However, once the biological night is reached, frontal low EEG activity and sleepiness steeply increase, while performance and well-being deteriorate concomitantly. Intriguingly, once the biological day is reached (*i.e.*, after around 31 h of continuous wakefulness), the values stabilize or even approach baseline levels, even though wakefulness is further extended (see also [22,35,36,37,52]). This daytime stabilization under high sleep pressure is most likely due to circadian arousal-promoting mechanisms that oppose high sleep pressure levels during daytime [54].

While it is tempting to assume that all these measures are closely correlated, underlining evidence is mixed so far. Most studies focused on the relationship between subjective and objective sleepiness, assessed under rising and high sleep pressure (previously reviewed for the Karolinska Sleepiness Scale [55]). However, under high sleep pressure, people react differently (but intra-individually stable) according to the domain of measurement (e.g., domains such as self-evaluation of sleepiness, cognitive processing capability or sustained attention performance [56]). Thus, given the same person, subjective and objective sleepiness might not be affected to the same extent by high sleep pressure. This might hamper a striking correlation between differential measures assessed under such conditions.

### 2.4. Neuronal Underpinnings of Sleep and Wakefulness and the Role of Adenosine

#### 2.4.1. Adenosinergic Regulation of Sleep Homeostasis

Sleep homeostatic mechanisms in the brain have been associated with the increase and decrease of substances, so-called sleep-factors, in widespread cerebral networks (e.g., adenosine, nitric oxide, tumor necrosis factor α, brain-derived neurotrophic factor or interleukin 1 [9]). Here, the focus will be on evidence underlining the role of adenosine and its metabolism. Its important role in human sleep-wake regulation is underlined by the world-wide common use of the non-selective adenosine antagonist caffeine [7].

##### A Role of Adenosine in Sleep Homeostasis—Implicated Brain Regions

**Figure 4:**
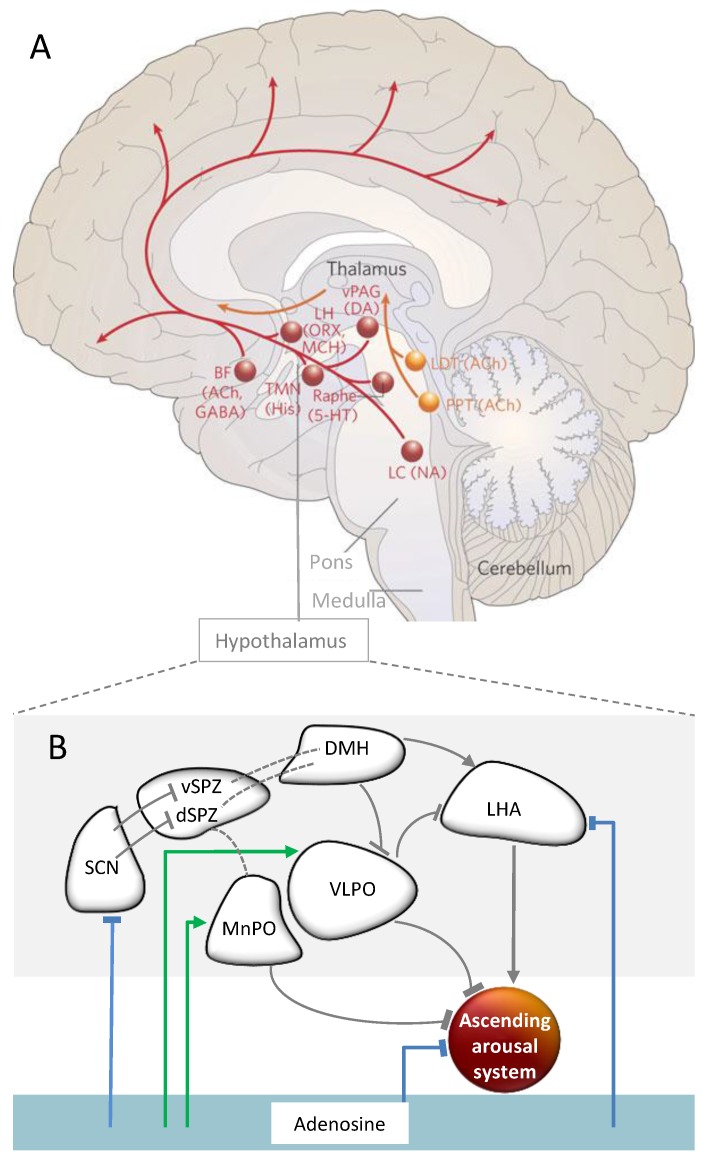
Hypothalamic regulation of the ascending arousal system and the impact of adenosine. (**A**) The ascending arousal system. One of the main pathways (**red**) activating the cortex arises from neurons in the monoaminergic cell groups, including the locus coeruleus (LC) containing noradrenaline (NA), the dorsal and median raphe nuclei containing serotonin (5-HT), the A10 cell group containing dopamine (DA), and the tuberomammillary nucleus (TMN) containing histamine (His). This pathway receives contributions from peptidergic neurons in the lateral hypothalamus (LHA) containing orexin (ORX) or melanin-concentrating hormone (MCH), and from basal forebrain (BF) neurons that contain γ-aminobutyric acid (GABA) or acetylcholine (Ach). The red pathway activates the cerebral cortex to facilitate the processing of inputs from the thalamus. **Orange** lines represent input to the thalamus originating from cholinergic (ACh) cell groups in the upper pons, the pedunculopontine (PPT) and laterodorsal tegmental nuclei (LDT). Figure and legend modified from a previous work [81]. (**B**) Hypothalamic and adenosinergic arousal modulation. The SCN innervates by GABA-ergic neurons the ventral supraventricular zone. The latter projects to the dorsomedial hypothalamus (DMH), in turn providing glutamatergic input to the orexin-containing neurons in the lateral hypothalamus (LH). These finally target the locus coeruleus (LC), a major player in the ascending arousal system [87]. The ascending arousal system is inhibited by GABA-ergic input of the ventrolateral and median preoptic area (VLPO and MnPO), and in part by adenosine. Adenosine acts inhibitory (**blue** lines) via A_1_ or disinhibitory (**green** lines) via A_2A_ receptors on several structures within this network. Striped lines show neural projections of which implicated neurotransmitters are currently unknown.

The nucleoside adenosine is intra- and extracellularly ubiquitous in the central nervous system. It acts on sleep-wake regulation mainly via its widely distributed inhibitory A_1_ receptors [7,8]. In animals, adenosine levels increase in several brain areas during extended wakefulness, and decrease during recovery sleep from SD. Moreover, adenosine inhibits arousal and induces sleep, modulated by receptors in the basal forebrain [57,58,59,60]. Evidence suggests further inhibitory influences on other structures crucially involved in arousal promotion (for an overview, see Figure 4a) such as the tuberomamillary nuclei (TMN), or orexin containing neurons in the lateral hypothalamus (LH; [8]). Conversely, adenosine has an excitatory influence via A_2A_ receptors in the sleep-promoting neurons in the ventrolateral preoptic area (VLPO) of the hypothalamus (Figure 4b; [61]). In sum, adenosine appears to be a powerful modulator of arousal-promoting brain structures.

##### Why Does Adenosine Increase with Time Spent Awake? Contributions of Its Metabolization

Adenosine is the end-product of the hydrolysis of adenosine triphosphate, the so-called “energy currency” [8]. Consequently, it has been related to the energy consumption of a cell [8]. However, an increase and decrease of adenosine, linked to sleep homeostasis, is not necessarily or exclusively due to increased or decreased energy demands. It can also strongly depend on adenosine metabolization and transport. Extracellular clearance of adenosine is mostly regulated via nucleoside transporters [62] or ecto-adenosine deaminase [7]. Intracellularly, adenosine is converted by adenosine kinase, or metabolized by adenosine deaminase (ADA) to inosine [7]. The ADA-dependent degradation plays a presumably crucial role under conditions of high adenosine concentrations [62].

There is evidence that adenosine degradation plays a role in sleep wake-regulation. For instance, the activity of several adenosine metabolizing enzymes shows a diurnal rhythm [63]. During the active phase, ADA activity has been observed to peak in the VLPO, while exhibiting troughs in the basal forebrain. Additionally, pharmacological inhibition of ADA leads to a rise in extracellular adenosine and prolongs NREM sleep [64,65,66]. Moreover, Franken *et al.* demonstrated that a region which encodes ADA in mice is associated with the rate of NREM sleep need accumulation [67]. However, ADA activity remained unchanged after SD in several sleep-wake regulatory brain areas, such as the LC, TMN, VLPO and basal forebrain [63].

In humans, the single nucleotide polymorphism rs73598374 has been linked to differences in enzymatic activity in blood cells [68,69]. Compared to G/G-allele carriers, the catalytic ADA activity is reduced in G/A-allele carriers by around 20%. Enzymatic activity in A/A-allele carriers is unknown so far. Longer SWS and higher EEG power in the slow-wave range during NREM sleep was observed in G/A- compared to G/G-allele carriers in baseline [70,71] and recovery nights from SD [71]. An epidemiological study revealed higher sleep efficiency, but not a longer duration of SWS, in G/A-allele carriers [72]. Yet, a higher EEG power in heterozygous individuals in the delta range during SWS was confirmed, which was, however, limited to occipital derivations [73]. Furthermore, higher subjective sleepiness in G/A- compared to G/G-allele carriers over 40 h [12,71] underlines a potential higher sleep pressure in heterozygous individuals. However, the results regarding genotype-dependent differences in vigilance performance is less consistent [12,71,74], potentially due to small sample sizes, and differential statistical methods.

Overall, the literature suggests that the adenosinergic system, including built up, degradation, transport and receptors (an overview about receptors is available [75]), strongly contributes to changes in physiology and behavior according to time spent awake. The existing evidence indicating an interaction of adenosinergic with circadian mechanisms will be summarized next, after a brief description of the pathways and neurotransmitters involved in circadian sleep-wake promotion.

#### 2.4.2. Pathways of Circadian Arousal Promotion

The SCN has a central role in the regulation of circadian rhythmicity. It has often been labelled as the director of an orchestra of circadian rhythms ticking in most cells of the body (e.g., [76,77]). SCN lesions in animals [29] and humans [78,79] indicate that the SCN is not only crucially involved in the timing of sleep and wakefulness, but also in its consolidation (reviewed in [80]). However, it has to be noted that all lesions might have involved a destruction of SCN adjacent areas [29,80].

The SCN receives light-dark information via the retinohypothalamic tract [30]. Downstream from the SCN, circadian arousal promotion during daytime is most likely mediated via several interfaces, including the dorsomedial hypothalamus (DMH) and orexinergic neurons in the LH (Figure 4b). The latter have been shown to be crucially important to consolidate wakefulness [81]. They target the noradrenergic neurons of the locus coeruleus (LC; [29]). Together with other projections (Figure 4a), the LC provides excitatory input to a widespread cortical network (Figure 4a; e.g., [81,82,83,84,85,86,87]).

During the biological night, circadian arousal promotion is reduced. The circadian phase information is, again via several interfaces (Figure 4b), transduced to arousal-inhibiting brain structures [29]. Particularly important in arousal inhibition are sleep-active neurons of the VLPO. They inhibit via GABA-ergic input not only orexinergic LH neurons, but also nearly all brainstem structures, mediating arousal, such as the TMN, raphe nuclei, pedunculopontine and laterodorsal tegmental nuclei and the LC [81]. In turn, the activity of the VLPO is inhibited by the ascending monoaminergic projections, for instance, from the LC, and by GABA-ergic input from the DMH. This forms a reciprocal system between arousal promoting and reducing brain areas [29,88].

### 2.5. Interactions of Circadian and Sleep Homeostatic Mechanisms

#### 2.5.1. The Genetic Clockwork and Sleep Homeostasis

Evidence for an interaction between homeostatic and circadian processes has been reported within the genetic clockwork. In simplified terms, this self-sustained circadian feedback loop consists of several positive and a negative components [3]. The positive components include the coupling of the transcription factors CLOCK and BMAL1, or NPAS2 and BMAL1, which drive the transcription of *Period (Per1, Per2)* and *Cryptochrome (Cry1, Cry2)* genes. After translation, PER-CRY protein complexes translocate back to the nucleus to inhibit their own transcription via inhibition of the complex CLOCK/BMAL1 and NPAS2/BMAL1 (negative component of the feedback loop). Through posttranslational protein modifications, this inhibition is removed and a new cycle can begin [3]. Furthermore, the network comprises a range of additional feedback loops between core genes, interactions with several modulators during the processes of transcription and translocation, and also posttranscriptional and posttranslational modifications [3,89].

So far, it is not fully understood, how sleep homeostatic changes impact on the circadian feedback loop. Based on knock-out studies, we know that a lack of *Bmal* [90], *Npas2* [91], *Per1* and *Per2* [92], as well as *Cry1* and *Cry2* [93,94] is associated with changes in delta power during recovery sleep from SD. Furthermore, messenger ribonucleic acid (mRNA) levels of clock genes, such as *Per2* or *dbp* (D-site binding protein), in the cerebral cortex and other tissues are altered after SD [95]. Interestingly, in non-sleep deprived animals as well, *Per2* expression varies in the forebrain according to EEG delta power, while expression in the SCN appears to be independent of prior sleep-wake history [96,97,98].

Similarly, there is first evidence that adenosine might exert an influence on the circadian clockwork. It has been shown that A_2A_ receptor antagonism and knock-out of an equilibrative adenosine transporter (equilibrative nucleoside transporter 1 [ENT1]) is linked to blunted *Per2* expression in the striatum, but not in the SCN [99]. Yet, to our knowledge, a direct link between the sleep-homeostatic regulation of adenosine (*i.e.*, its regulation according to time spent awake) and the genetic circadian machinery, has not yet been explored. Franken, in a recent review, suggested that sleep homeostatic mechanisms might affect *Per2* expression via corticosterone-bound glucocorticoid-receptors, changes in brain temperature, metabolism, cytokine levels, altered DNA binding of NPAS2 and BMAL1, or the redox state [3]. Interestingly, the redox state has been associated with the excitability of SCN neurons through modulation of several potassium (K^+^) channels [100].

The circadian changes in membrane excitability and neuronal firing patterns of the SCN depend on the interplay of a number of ionic factors, such as K^+^ and Ca^2+^ currents [101]. They translate the time signal from the molecular clock into electrophysiological circadian fluctuations, characterized by a higher frequency and a higher input resistance during the subjective day compared to the subjective night [102]. How these fluctuations change in the SCN according to sleep pressure will be summarized in the next section. Furthermore, we will briefly describe other brain regions potentially implicated in the interaction of the adenosinergic modulation of sleep pressure and circadian mechanisms.

#### 2.5.2. Brain Regions and Substances Mediating the Interaction

In addition to research at the molecular level, electro-physiological studies underline an interaction between circadian and sleep homeostatic mechanisms amongst others directly in the SCN. Evidence indicates that firing rates of SCN-neurons are reduced during NREM as compared to REM sleep [103]. Additionally, SCN-activity correlates negatively with sleep pressure [103] and the amplitude of SCN activity is reduced after SD [4]. In humans, a differential modulation of the SCN according to sleep pressure is underlined by BOLD activity assessed by functional magnetic resonance imaging (fMRI). In line with the results derived from animal studies, BOLD activity in a hypothalamic region, putatively encompassing the SCN, was negatively associated with SWA [5].

Furthermore, SD studies, challenging sleep homeostatic mechanisms, indicate a role of adenosine in circadian amplitude and timing. A sleep-loss induced reduction of the SCN response to light [104,105] could be reinstated by treatment with the adenosine antagonist caffeine [105]. Thus, it has been suggested that adenosinergic A_1_ receptors might be involved in a sleep homeostatic modulation of the activity of the main circadian pacemaker [105]. So far, how this modulation is influenced by circadian and light-dependent variations of adenosine in the retina [106,107], and its relation to adenosinergic neurotransmission in the retinohypothalamic tract [108], has yet to be studied.

In mice [105] and human cells [109], caffeine treatment lengthened the circadian period under normal waking conditions. In humans, an interaction of the adenosinergic with the circadian timing system is indicated by a melatonin suppression [110] and a phase delay in response to acute caffeine administration [109], and is further underlined by our observation of an ADA-genotype dependent shift in the rise of melatonin [12]. Together with evidence from the animal domain [105,111,112,113], these findings suggest adenosinergic mechanisms underlying a circadian phase shift in response to heightened sleep pressure levels due to partial [13,114] as well as total SD [34].

An integration of circadian and sleep homeostatic inputs is also reasonable in the orexin-containing LH [115]. Orexin-levels show a circadian rhythm, but are also influenced by sleep homeostatic mechanisms [116]. The impact of sleep pressure might be regulated by adenosinergic A_1_ receptors in the LH. Adenosine inhibits orexinergic LH activity [117,118], which stabilizes the state of wakefulness and prevents sudden transitions to sleep [81,119]. In humans, such a promotion of wakefulness is particularly important in the evening hours [27], when sleep pressure is at high levels. During this time window, we observed that the ADA-polymorphism was associated with systematic differences in wake-promoting strength [49], as indicated by a lower sleep efficiency [27].

Finally, given the widespread projections of the ascending arousal system, indirectly connected to the SCN (see Figure 4), and the distribution of adenosinergic receptors all over the brain, the integration of circadian and homeostatic signals is reasonable in various brain regions at the single neuronal level. It has been shown in rats that the density of adenosinergic A_1_ receptors in the basal forebrain is upregulated in response to SD [120]. Similarly, in humans, A_1_ receptor binding is increased after SD in several cortical and subcortical regions [121]. Interestingly, the binding potential of these receptors has been proposed to show a circadian pattern in the cerebral cortex in animals [122]; however, this finding needs replication under constant lighting conditions.

Overall, the evidence strongly supports an interaction between sleep homeostatic and circadian mechanisms based on complex and widely distributed neuronal mechanisms. While we focused here on evidence regarding adenosinergic mechanisms, it should be noted that research has already identified a range of other sleep-factors (for overview, see [9]). In all likelihood, these not only are implicated in the increase and decrease of homeostatic sleep pressure, but also contribute to the mutual influence of sleep homeostatic and circadian processes.

## 3. Working Memory

As mentioned in Section 2.3.2, sleep-wake regulation crucially impacts cognitive performance. Circadian and sleep homeostatic variations have repeatedly been reported for several cognitive domains (see [123] for overview). The interaction of sleep-wake-regulatory mechanisms can be nicely observed during total SD: Over the course of a normal 16 h waking day, performance stays at rather stable levels, but steeply decreases once the biological night is reached. During the following day, cognitive performance stabilizes at a certain level or even improves again, in spite of rising sleep pressure levels during constant wakefulness (see Figure 3). This stabilization or recovery of performance during the day after a night of sleep loss is most likely due to circadian wake-promoting factors opposing high sleep pressure [54].

So far, research has focused mainly on the impact of SD on cognitive performance, whereas pure circadian variations, independent of sleep pressure, have been less extensively studied. Sleep pressure has been shown to affect several cognitive domains ranging from basic attentional processes, such as vigilance, to higher-order cognitive functions, such as working memory or decision-making [123]. The latter processes have been proposed to be particularly vulnerable to SD because of their dependence on the prefrontal cortex (PFC) [124]. The hypothesis was based on EEG ([125], and see also [43,51] for further evidence) and early neuroimaging studies [126,127], underlining an influence of homeostatic sleep pressure, particularly in frontal brain areas. In the following, we focus on evidence about sleep-homeostatic influences on working memory (WM), a complex cognitive process relying on the PFC’s integrity [128]. After a brief definition of WM and its neuroanatomical basis, we will discuss a potential role of adenosine in mediating the influence of sleep pressure and finally consider the current evidence of circadian modulation of WM and its modulation by sleep pressure.

### 3.1. Working Memory at a Conceptual Level

The main process characterizing WM performance is generally considered as the successful manipulation of information in a kind of short-term storage. Importantly, WM is distinct from short-term memory in that it not only refers to a brief storage of information, but also to its manipulation. Similarly, in animal research, WM is understood as “... a representation of an object, stimulus, or spatial location that is typically used within a testing session, but not between sessions, to guide behavior” [129]. Irrespective of a limited storage capacity to a specific “magical” number of items [130,131], WM performance can be trained successfully by practicing executive aspects of WM [132,133,134]. Executive aspects refer to processes apart from storage, for instance, monitoring and updating of information, inhibition of prepotent responses and mental set shifting (e.g., [135,136]).

The currently best-known conceptualization of WM was originally published by Baddeley and Hitch first in 1974 [137], and has been continuously refined ever since. According to this multicomponent theory, the WM system is comprised of several modules [138]: At least two capacity-limited storage modules, termed phonological loop and visual-spatial sketchpad, are assumed to store information in a modality specific manner over short terms. These storage modules are linked to an executive control system. The central executive regulates the manipulation of information within the storage modules. It is assumed that it controls the focus and the division of attention, and guides decision-making and switching between tasks. Thus, it is central for processes commonly labeled as executive functions [138].

Mirroring the diversity of WM processes, there is a wide range of tasks assessing WM functions. For instance, the WM span tasks are classically used to measure capacity limits of WM (see [139] for a detailed discussion on reliability and validity). Serial addition tasks serve to assess speed of processing and updating performance in the short-term storage [140]. Updating information in the short-term storage, and particularly underlying cerebral correlates, were often deduced by performance in the n-back task (see [141] for discussion). Furthermore, there are specific tasks focusing on executive aspects of WM, such as the stroop task or the go/no-go paradigm to assess inhibition capability (e.g., [142]). Generally, the use of a particular task depends on its validity, reliability, and feasibility to implement it in a specific setting, such as MRI or conditions of repeated measurements in FD, SD and NP studies. In animal research, WM performance is often assessed by so-called delay response paradigms in primates [143], and the Morris water maze [144] or radial arm maze [145] in rodents.

### 3.2. Neuronal Underpinnings

#### 3.2.1. Brain Activity Patterns

As reviewed extensively by others [136,146,147,148,149,150,151,152], brain activity during a WM task typically involves widespread networks, ranging from prefrontal areas to parietal regions as well as the occipital lobe. The temporal patterns of the activity distributions appear to fit well to the conceptual models of WM: Broadly speaking, prefrontal areas, reminiscent of the central executive, control activity in sensory regions, representing modality specific storage modules [150].

Generally, persistent activity in lateral prefrontal neurons mirrors top-down control of those regions, which maintain sensory information. The lateral PFC presumably exerts its top-down control by both active promotion of relevant information and active suppression of irrelevant information [150]. It has been suggested that the ventrolateral part of the PFC mediates a controlled access to memory contents and their maintenance [148], while the dorsolateral region appears to be more implicated in the organization of WM contents into higher-order units of information, so-called chunks [146].

To regulate interference reduction, the pre-supplemental motor area appears to be particularly important [152]. This area has also been proposed to play a role in the capacity limits of WM, and linked to the limits in selective attention [153]. A further limiting factor for capacity is activity in more posterior parietal areas, which are crucially involved in formation and maintenance of information [150,153].

Finally, subcortical areas, such as the striatum or cerebellum are involved in successful WM performance, for instance, in the suppression of irrelevant information [150] or maintenance of information and guiding attention [149]. Furthermore, the maintenance of information, particularly in spatial and relational tasks, has repeatedly been linked to the hippocampus and related brain areas in the medial temporal lobe [154,155].

#### 3.2.2. Neurotransmitters and Neuromodulators

Numerous neurotransmitters are involved in the regulation of WM performance. Aside from evidence underlining a role of acetylcholine, norephinephrine or serotonin in the PFC [156], the presumably largest body of research targets the role of dopamine in the PFC [157,158]. The effects of dopamine are mirrored in an inverted u-shaped function, such that a deregulation in any direction has a detrimental impact on performance [159]. In humans, strong evidence for a dopaminergic modulation of WM arises from impairments of WM functions following pathophysiological changes in the dopaminergic system (for instance, in schizophrenia [160]).

Intriguingly, the adenosinergic system also plays a role in WM performance modulation under conditions of normal sleep-wake cycles. As adenosine receptors are expressed in brain areas, characterized by dopaminergic innervation (e.g., in the cortico-striatal pathways), they are optimally suited to provide a basis for the interaction between these neurotransmitter systems [161]. Numerous studies have shown an antagonistic interaction particularly of A_2A_ and dopamine D_2_ receptors in the striatum, and there is also evidence for a similar pattern between A_1_ and dopamine D_1_ receptors [161]. In rodents, WM performance was enhanced after acute injection of an A_1_ receptor agonist into the hippocampus [162,163]. Similarly, overexpression of adenosine kinase, an enzyme implicated in clearance of adenosine, was linked to a decrease in WM performance [164,165]. Implants of adenosine releasing cells, located proximal to the hippocampus, restored these WM deficits [164]. However, while these studies indicate a beneficial effect of adenosine availability on WM performance, there is also evidence pointing in the opposite direction. For instance, injection of an A_2A_ receptor antagonist is linked to improvements in WM performance [166]. In line with this result, WM performance was reduced in rats overexpressing A_2A_ receptors in the cortex, hippocampus and cerebellum [167]. Knock-out of a gene encoding ecto-5′-nucleotidase, an enzyme converting adenosine monophosphate into adenosine, was associated with increased WM performance [166,168]. Furthermore, A_2A_ receptor knock-out in the entire brain [169], and in particular in the striatum [170], has been linked to enhancements in WM performance.

Differences between the mentioned studies in the target receptors and their different distribution throughout the brain (see Section 2.4.1) might account for conflicting results, together with divergences in the WM tasks used. Moreover, in the light of a detrimental influence of both artificial enhancement and reduction of the adenosinergic tone, Singer *et al.* suggested that a “physiological adenosine concentration” appears to be optimal to maintain a stable neural network underlying WM, including glutamatergic and dopaminergic networks [171].

In humans, a reduction of the adenosinergic tone appears beneficial for performance under pathophysiological conditions [172]. However, in healthy adults, performance did not significantly change after an administration of the adenosine antagonist caffeine, even though differences in underlying brain activity patterns were observed [173,174,175]. Notably, the latter studies were conducted under normal waking conditions. As summarized below, performance is indeed affected by prolonged wakefulness, an effect potentially modulated by adenosinergic mechanisms.

### 3.3. Impact of Sleep Loss

As summarized by Lim and Dinges (2010) in a meta-analysis, WM performance is robustly affected by sleep loss [176]. However, it is still a matter of debate in which of the various processes constituting WM performance these decreases specifically occur [177]. According to the so-called vigilance hypothesis [176], decrements in performance might be traced back to a general decline in basic attentional processes, such as arousal, required to perform in a WM task. Tucker *et al.*, for instance, disentangled executive from non-executive WM components and showed that specifically the latter were affected by extended wakefulness [178].

In parallel, sleep loss-related declines in WM have been proposed to be due to their particular dependence on activity in the PFC. Harrisons and Horne [124] suggested that the PFC, continuously challenged during wakefulness, is specifically sensitive for the effects of SD. This so-called neuropsychological hypothesis [176] is underlined by a predominance of delta and theta power EEG in frontal areas during recovery sleep from SD [43]. Additionally, in animals, the wake-dependent increase in adenosine has been specifically observed in the basal forebrain, located frontally [57]. Furthermore, the up-regulation of human A_1_-receptors after 24 h SD has been reported to be most pronounced in the orbito-frontal cortex [121]. In further support for the neuropsychological hypothesis, Drummond *et al.* showed that performance in the inhibition of prepotent responses, a specific executive aspect of WM, was impaired by SD, while the general ability to correctly respond to frequent trials was not affected [179]. A similar specific effect of high sleep pressure has been reported regarding the executive WM component of switching [180].

However, several studies report stable levels of WM performance, specifically for higher-order executive functions, over the course of SD. Roughly a decade ago, it was even considered as “prevailing view in SD research (…) that high-level complex skills are relatively unaffected by SD(…)” [124], p. 236. This view was based on the idea that higher-order cognitive tasks generate a kind of motivation or interest, which leads to compensatory effort to perform well, even under high sleep pressure [124].

Accordingly, neuroimaging studies investigating the impact of sleep loss on WM-related brain activity revealed a complex pattern of increases and decreases in several brain regions [181]. Compared to baseline, activity decreases have been observed after sleep loss in fronto-parietal brain areas and associated with declines in several aspects of WM performance [182,183,184,185,186,187]. The maintenance of stable WM performance in sleep deprived states has been traced back to compensatory increases at the brain activity level in frontal, anterior cingulate and thalamic areas [182,184,186,188]. One factor modulating compensatory increases has been proposed to be task complexity: Better performance after SD was observed in the more complex tasks and proposed to be related to increases in prefrontal and thalamic activity [182]. Furthermore, it has been observed that individuals highly differ in compensatory brain activity patterns. These variations presumably underlie stable inter-individual differences in vulnerability to sleep loss at the behavioral level [189].

#### Do Adenosinergic Mechanisms Play a Role in Sleep-Pressure-Related WM Deficits?

As briefly mentioned above, the blockade of adenosinergic receptors by caffeine has previously not been associated with enhanced WM performance when studied during a normal waking day [21,174,175]. However, caffeine exerted a positive effect on performance in several executive functions when administered after 64 h of SD [190]. Additionally, caffeine ameliorated wake-dependent decreases in short-term memory performance only after a certain sleep pressure level was reached [21]. Thus, a blockade of adenosinergic receptors appears to be beneficial for WM under conditions of sleep-pressure-related performance troughs.

Furthermore, there are indications that genetic variations in the adenosinergic system impact on WM performance according to sleep pressure levels. Under conditions of sleep restriction, knockout of neuronal adenosine A_1_ receptors was associated with a reduction of WM performance compared to performance of wild phenotype animals [191]. Furthermore, in humans, WM performance was differentially regulated according to the ADA-polymorphism rs73598374. We observed that sleep-pressure-dependent performance modulations, specifically in executive aspects of WM, were evident in G/A- but not in G/G-allele carriers [12]. Moreover, compared to G/G-allele carriers, G/A-allele carriers benefited more in WM performance from REM sleep duration in the early morning hours [49].

Together, these studies suggest that a behavioral variation following a dampening of adenosinergic effects, either due to receptor blockade, receptor expression, or differential metabolization, might be more likely observed under conditions of a high adenosinergic tone and a concomitant high sleep pressure level.

### 3.4. Circadian Modulation

Finally, it is important to consider that the impact of sleep loss on WM performance is dependent on circadian phase. The present evidence indicates that partial aspects of WM performance deteriorate at night, such as processing speed, focused attention and short-term memory functions [16,17,18,19,20,21]. However, higher executive functions remain rather stable during nighttime, as, for instance, inhibition [22] or planning performance [23]. Correspondingly, Monk reported a negative correlation of the circadian variation in cortisol and WM speed, while WM accuracy was not significantly associated [192].

Intriguingly, under conditions of sleep loss and concomitant high sleep pressure levels, higher-order executive functions have been observed to decrease at night. This pattern is not simply due to a rise in sleep pressure, as performance stabilized or increased again when wakefulness was extended to the following day [22,23]. Thus, the impact of sleep pressure is enhanced during the night, but counteracted during the day. This interaction of sleep homeostatic and circadian mechanisms was also generally shown in tasks assessing more basic functions, but not in a consistent manner [16,17,18,19,20,21]. These inconsistencies are most likely due to study designs, in which wakefulness was restricted to less than 16 h. Under these conditions, sleep pressure levels might not be high enough to exert a clear-cut influence at night [17,20,21].

**Figure 5:**
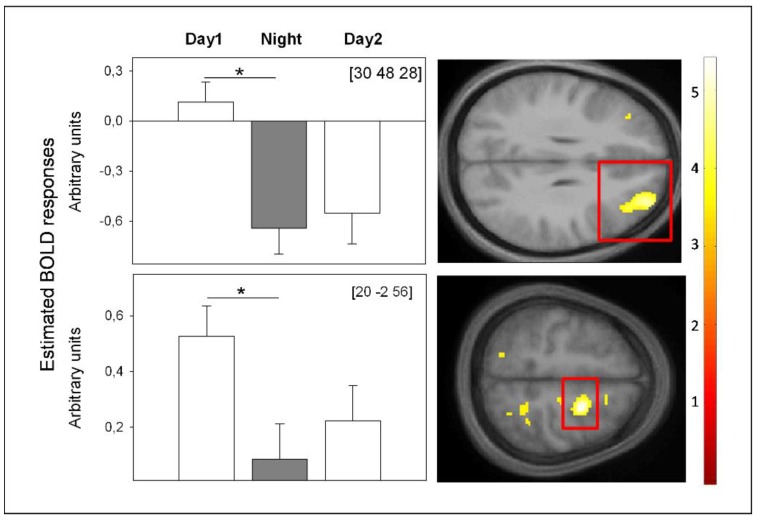
BOLD activity underlying successful WM during total SD. BOLD activity was assessed during hit targets in a 3-back task after 13 h (Day 1), 21 h (Night) and 37 h (Day 2) of continuous wakefulness in constant posture and lightning conditions. From a circadian perspective, these time windows encompass maximal promotion of wakefulness (Day 1, Day 2) and sleep (Night). Left: Significant changes in estimated BOLD responses in frontal brain regions. Right: Location of significant changes in estimated BOLD response, highlighted by red rectangles on mean structural imgages. * p < 0.01; *p*-value at peak-level after family-wise error correction (FWE), applied on a mask encompassing brain regions typically underlying n-back performance according to [146]. We did not find any other significant BOLD activity changes. Coordinates (*x*, *y*, *z*) are expressed in mm in the Montreal Neurological Institute (MNI) space, (unpublished data) [193].

Recently, we assessed cerebral correlates of higher-order WM performance during both a 40-h SD protocol and a 40-h NP. Similar to Chee *et al.* (2006) [183], the data revealed that performance and underlying frontal BOLD activity decreased specifically under SD from day to nighttime (Figure 5). When wakefulness was extended to the evening of the next day, we observed performance increases from nighttime to the second day and no significant concomitant changes in frontal BOLD activity (Figure 5). The latter indicates stabilization at the cerebral activity level despite of rising sleep pressure levels, which might be either due to a floor effect in BOLD activity or to the switch in circadian phase from night to daytime. Interestingly, during the second day, a superior frontal area (*x* = 20; *y* = −2; *z* = 56), underlying successful WM performance, was functionally connected to a region in the hypothalamus. Together, these data underline the interaction of circadian and homeostatic mechanisms at the BOLD activity level. They indicate that high sleep pressure, specifically at night, is detrimental for frontal brain activity, underlying successful WM performance. As soon as daytime is reached, circadian alerting signals of hypothalamic origin might support WM underlying brain mechanisms such that brain activity does not further decline, despite rising sleep pressure.

In sum, the emerging picture suggests that the more basic processes required for WM decrease during nighttime. These nighttime troughs are pronounced under high sleep pressure, and can only be observed under these adverse conditions in higher-order executive functions. Under low sleep pressure, complex tasks might trigger motivational resources that help to overcome circadian nighttime troughs in cognitive performance [124]. Alternatively, compensatory brain mechanisms might operate in a more successful manner when the implicated network is more widespread, as typical for more complex cognitive processes [189]. Such compensatory mechanisms might contribute to a vulnerability to sleep loss at night.

## 4. Conclusions

The empirical evidence, summarized above, leaves no doubt that sleep-homeostatic and circadian mechanisms do interact. Differential methodological approaches not only revealed possible interfaces of sleep-homoeostatic processes and the circadian timing system, but also indicate that the influence of sleep pressure is differentially modulated according to circadian phase. Given the possibility of an entrainment to cyclic environmental changes, such an interaction appears to be adaptive: Periods of sleep, necessary for survival [194], are more intensively favored during times when the environmental light-dark situation favors rest. The other way around, the impact of homeostatic sleep propensity is counteracted as long as environmental light conditions allow optimal wakefulness (e.g., successful foraging during daylight in diurnal species).

At present, studies indicate that there is a role for adenosine in sleep homeostasis and in the interaction with circadian mechanisms. However, the particular adenosinergic contributions to the effects of time spent awake on several levels of physiology and behavior remains to be specified. Future research might focus not only on interactions with other sleep factors, but also on inter-individual differences due to ageing and genetic predispositions. A further open question concerns differences between acute and long-term changes in adenosinergic mechanisms. So far, in humans, the sparse empirical evidence lets us assume adaptations to variations in the adenosinergic systems over long terms [195]. However, the temporal dynamics of this process and the interaction with inter-individual differences are unknown, as, for instance, differences in adenosine receptors and caffeine sensitivity [196].

Research targeting these questions might also be applied to the effects of adenosine on cognitive performance and underlying cerebral correlates. The studies summarized above suggest a beneficial effect of adenosinergic blockades on WM performance, when assessed under conditions of sleep-pressure-related performance declines. Cerebral correlates of adenosinergic benefits under high sleep pressure remain to be elucidated, controlled for a confounding influence of adenosine on BOLD activity patterns [197]. Similarly, the positive effects of circadian wake-promotion after a night of sleep loss have to be studied in more detail, not only in the domain of WM, but also in other cognitive domains. Finally, sophisticated analyses on neuroanatomic and neuropharmacological conditions of circadian wake-promoting activity at the individual level might have implications for the frequently encountered performance errors during night and shiftwork.
